# Comparative outcomes of endodontically treated teeth versus dental implant-supported prostheses: a systematic review

**DOI:** 10.2340/aos.v84.43871

**Published:** 2025-06-24

**Authors:** Miguel Fernando Borda, Salomón Páez-García, Luisa Fernanda Murcia, Luis Carlos Venegas-Sanabria, Miguel Germán Borda

**Affiliations:** a BGR Dental, West Palm Beach, FL, USA; b Semillero de Neurociencias y Envejecimiento, Ageing Institute, Medical School, Pontificia Universidad Javeriana, Bogotá, Colombia; c Research Department, Hospital Universitario Mayor-Méderi, Universidad del Rosario, Bogotá, Colombia; d Rosarist Institute for the Study of Aging and Longevity, Universidad del Rosario, Bogotá, Colombia; e Centre for Age-Related Medicine (SESAM), Stavanger University Hospital, Stavanger, Norway; f Department of Neurology, Clínica Universidad de Navarra, Pamplona, Spain

**Keywords:** root canal therapy, endodontics, dental implant, single tooth implant, outcomes assessment

## Abstract

**Objective:**

The objective of this study was to compare the clinical and patient-related outcomes of root canal therapy (RCT) and dental implants (DIs) in managing severe tooth damage, supporting evidence-based treatment decisions.

**Material and methods:**

A systematic review was performed in accordance with PRISMA (Preferred Reporting Items for Systematic Reviews and Meta-Analyses) guidelines. Six databases were searched: Cochrane Library, Embase, Medline, LILACS, Scopus, and Web of Science. Studies comparing clinical and patient-reported outcomes in adult patients treated with either RCT or DIs were included. The Joanna Briggs Institute Critical Appraisal Checklists were used to assess the risk of bias. Due to significant heterogeneity among studies, meta-analysis was not feasible, and findings were synthesised qualitatively. PROSPERO registration: CRD42024584113.

**Results:**

Out of 1,876 initial records, 12 studies met inclusion criteria: 7 cohort studies, 4 case-control studies, and 1 randomised controlled trial. Most studies had low to moderate risk of bias. Both RCT and DIs demonstrated high survival rates, with RCT slightly outperforming DIs in terms of success. Failure rates ranged from 0.7% to 12.0%, with no significant differences between treatments. DIs were associated with a higher frequency of postoperative interventions and complications. Patient-reported outcomes such as pain, satisfaction, and quality of life (QoL) were comparable across both modalities.

**Conclusion:**

RCT and DIs both offer viable and effective solutions for restoring severely damaged teeth, with high survival and success rates and low failure rates. Both treatments were also associated with favourable patient-reported outcomes, including minimal pain, high satisfaction, and improved QoL. The decision between treatments should consider clinical factors, patient preferences, cost, accessibility, potential complications, and patient-centred outcomes. Shared decision-making is essential for optimal patient care.

## Introduction

When a tooth experiences significant structural damage because of decay, trauma, or infection, it can lead to serious dental conditions such as pulpitis and periapical pathology. These issues compromise the function and integrity of the affected tooth, potentially leading to complications like abscesses, systemic infections, or persistent pain if not promptly managed. Timely intervention is therefore essential to preserve oral health and prevent further deterioration [1–3].

Dental practitioners often face two primary options for managing such cases: preserving the natural tooth through root canal therapy (RCT) followed by appropriate prosthetic restoration, or extracting the tooth and replacing it with a standalone prosthetic solution, typically a dental implant (DI). RCT focuses on preserving the natural tooth by removing the infected or inflamed pulp tissue, disinfecting the canal system, and sealing it to prevent reinfection. In contrast, when preservation is no longer feasible, extraction and replacement with an implant-supported prosthesis provide an alternative that restores function and aesthetics.

Each treatment approach offers distinct benefits and limitations. RCT aims to preserve the natural tooth, maintaining its original structure and function. However, it may require multiple appointments, carries a risk of reinfection, and can weaken the tooth over time. In contrast, tooth extraction results in edentulism – a condition linked to adverse outcomes in older adults, including malnutrition, functional disability, cognitive decline, and poor self-rated health status [4]. DIs provide a highly effective alternative when tooth preservation is not feasible, offering excellent durability, and long-term success. Nevertheless, implant placement is more invasive, entails higher initial costs, and requires a longer recovery period, with potential complications such as implant failure or bone loss [5, 6]. As such, RCT and DIs differ markedly in terms of procedural complexity, healing time, cost, patient preference, and both clinical and patient-reported outcomes [7]. These factors are critical in guiding individualised treatment decisions.

Despite widespread use of both RCT and DIs, few studies directly compare their clinical and patient-reported outcomes using standardised definitions. Existing literature often focuses on survival or success rates without adequately addressing patient-centred metrics such as pain, satisfaction, or quality of life (QoL).

This systematic review aims to critically evaluate and compare the clinical outcomes and patient experiences associated with RCT and DI-supported prostheses. By synthesising current evidence, this study aims to guide clinicians in making informed treatment decisions in collaboration with their patients.

## Materials and methods

This systematic review followed the PRISMA (Preferred Reporting Items for Systematic Reviews and Meta-Analyses) guidelines (shown in Appendix2 B) [8].

### PICO Strategy

- **Population:** Adults (≥18 years) with compromised teeth due to pulpal or periapical diseases who required dental treatment.
- **Interventions**: Initial endodontic treatment, non-surgical or surgical endodontic retreatment.
- **Comparators:** Dental implants, including single or multiple dental prostheses.
- **Outcomes:** Described in the following subsections.

### Primary outcomes

- **Survival:** Defined as the presence of the tooth or implant in the patient’s oral cavity at the last documented follow-up.
- **Success:** Defined as the presence of the tooth or implant without any further intervention or planned intervention.
- **Failure:** Defined as the condition requiring retreatment or reconsideration of the treatment option.

### Secondary outcomes

- **Complications:** For endodontic procedures, this included flare-ups, accidental events during treatment, and post-placement issues. For implants, complications included unscheduled visits because of severe pain or swelling, surgical complications, bone loss, and esthetic or phonetic concerns.
- **Patient-reported outcomes:** Evaluated using patient-reported measures, including pain levels before and after treatment, satisfaction, and QoL assessed by instruments such as the Oral Health Impact Profile (OHIP) [9] in its various forms (OHIP-49, OHIP-14, or OHIP-19).

### Search strategy

A systematic search was conducted in six databases, Cochrane Library, Embase, Medline, LILACS (Latin American and Caribbean Health Sciences Literature), Scopus, and Web of Science, to identify studies comparing outcomes in adult patients (aged 18 years or older) who underwent either RCT or received DIs. A combination of key search terms was employed, including ‘root canal therapy’, ‘endodontic treatment’, ‘dental implants’, and ‘tooth implant’. The development of the search strategy, including the identification of appropriate search terms (both index terms and keywords) and the definition of the search structure (use of Boolean operators, truncation, and wildcards), was initially carried out by one author (LFM). To ensure the strategy’s accuracy and comprehensiveness, it was subsequently reviewed and refined by two additional authors (MGB and LCV). The final search was performed by LFM and completed in February 2025.

In addition, a supplementary screening was conducted in February 2025 using Google Scholar and BASE (Bielefeld Academic Search Engine), along with manual searches of the reference lists of selected studies and relevant systematic reviews. This complementary search did not identify any additional studies meeting the eligibility criteria for inclusion in the review.

### Selection criteria

The following inclusion criteria were applied:

1. Original research studies: interventional (randomised controlled trial) and observational studies (cohort, case-control, and cross-sectional studies).
2. Studies written in any language.
3. Studies that directly compare outcomes between RCT and DIs.

Reviews, dissertations conference proceedings, letters or poster abstracts were excluded. No publication date restrictions were applied. The complete search strategy is detailed in Appendix A.

### Screening and data collection

Following removing duplicate records, two independent reviewers (LFM, MGB) screened the titles and abstracts of identified studies using the RAYYAN web-based application [10]. Full-text articles of potentially eligible studies were then reviewed to ensure they met all inclusion criteria (MFB, LFM). Any discrepancies between reviewers were resolved through discussion, and if necessary, a third reviewer was consulted to achieve consensus (MFB). When multiple articles from the same study population met the inclusion criteria, preference was given to the article with the most comprehensive outcome data, the largest sample size, or the most recent publication date.

### Data extraction and analysis

Two independent reviewers used a standardised data extraction form to extract data (MFB, LFM). The extracted data included the principal author, publication year, country, type of study, collection dates, selection criteria, sample size, age, sex, treatment details, follow-up duration, outcomes, and key findings. Discrepancies in data extraction were resolved by discussion or consulting a third reviewer.

### Risk of bias assessment

Two authors (LFM and MFB) independently evaluated each study for risk of bias using the Joanna Briggs Institute (JBI) Critical Appraisal Checklists [11], which were appropriate for the study design. Based on the responses to the checklist items, studies were categorised as having low, moderate, high, or critical risk of bias. Any disagreements among reviewers during the risk of bias assessment were resolved through discussion or, if needed, by consultation with a third reviewer (LCV).

The study protocol was registered in PROSPERO (CRD42024584113).

## Results

**Figure 1:**
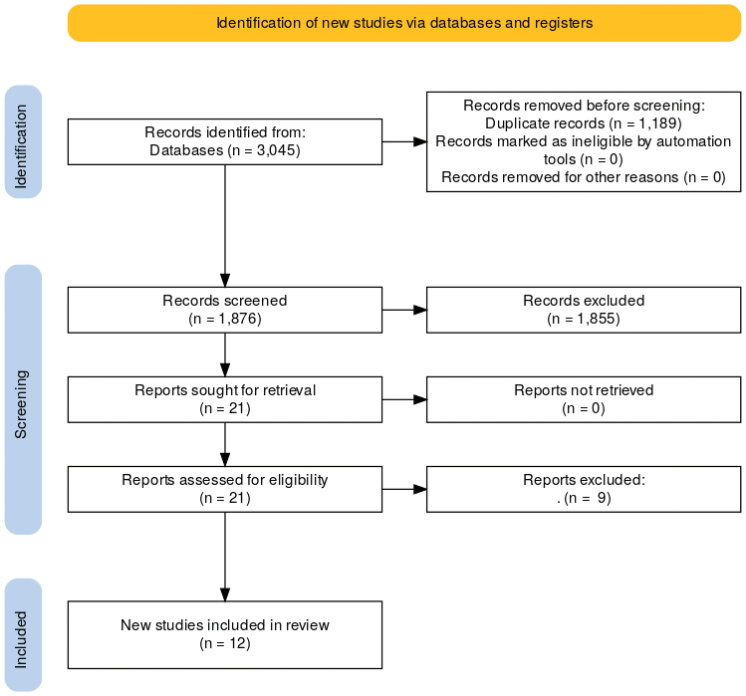
Flowchart of the systematic review process [21].

The database search initially identified 1,876 records. Following a review of titles and abstracts, 21 articles were selected for full-text review. Of these, 9 articles and 3 studies [12–14] were excluded because they reported data already presented in other studies included in this review; 5 studies [15–19] were excluded for evaluating interventions not aligned with the scope of this review or addressing outcomes beyond its predefined objectives; 1 article [20] was excluded due to being published in Persian, as a suitable translation could not be obtained for proper assessment (Figure 1).

### Risk of bias assessment

**Figure 2:**
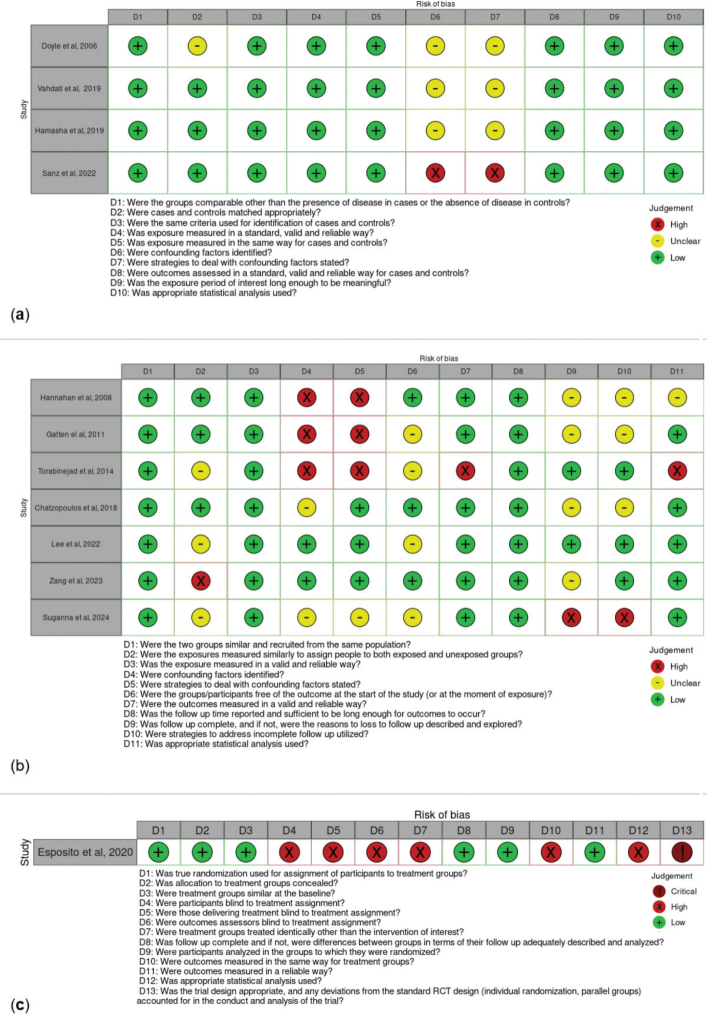
Risk of bias assessment for (a) case-control studies, (b) cohort studies, and (c) randomised controlled trial. The ROBVIS tool^®^ was used to generate this figure [22].

The results of the critical appraisal process are reported in Figure 2 [22]. Most of the included studies showed low to moderate risk of bias across the evaluated domains. Specifically, the studies presented some concerns in confounding adjustment [23–31] and follow-up process [27, 28, 30, 31].

**Figure 3:**
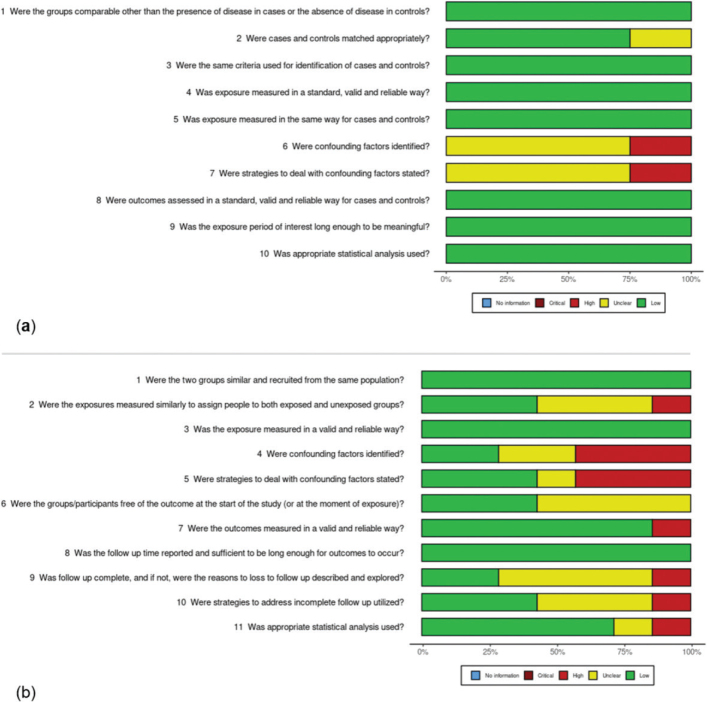
Global quality assessment for (a) case-control studies, (b) cohort studies. The ROBVIS tool^®^ was used to generate this figure [22].

In addition, in four studies [28, 30–32], it was unclear whether participants were free of the outcome at the start of the study or at the moment of exposure. The single randomised controlled trial included [33] showed a high risk of bias in several domains, most notably because of the substantial discrepancy between the estimated sample size of 240 participants (120 per group) and the final analysis, which was based on only 20 patients, as well as deviations from the intended intervention. A global summary of quality assessment is presented in Figure 3 [22].

Due to the considerable heterogeneity among the included studies, a meta-analysis was not performed. This heterogeneity was related to differences in study design, population characteristics, and the timing and methods used to measure both primary and secondary outcomes. These variations limited the comparability of results across studies, making it unfeasible to generate a pooled estimate. Consequently, a qualitative and descriptive synthesis of the findings was conducted.

### Overview of included studies

A total of 12 studies were included in the systematic review, comprising 7 cohort studies [27, 28, 30–32, 34, 35], 4 case-control studies [23–26], and 1 randomised controlled trial [33]. In general, the studies had a minimum follow-up time of 1 year, and considering all follow-up times for both treatments, the mean follow-up time was 2.8 years. They described non-surgical RCT followed by subsequent coronal restoration performed by dental students, graduate residents, or staff clinicians. Implants were generally described as single-tooth restorations supported by a single implant, surgically placed by staff or resident oral surgeons or periodontists, and restored by staff or resident prosthodontists. The studies compared the outcomes of RCT and DIs across the primary outcomes, including survival rates [23–25, 32, 34], success rates [23–25, 27, 31–33, 35], and failure rates [23–25, 27, 31, 33, 34], and secondary outcomes, including complications [23–25, 27, 30, 33] and patient-reported outcomes, such as QoL, measured by OHIP scores [23, 25, 30, 32] (Table 1).

**Table 1:** Summary of included studies.

Author, year	Country	Type of study	Collection dates	Intervention	Simple size	Age	Sex	Follow-up time	Survival rate (%)	Succes rate (%)	Failure (%)	Comp-lications	Patient-reported outcomes
Doyle, 2006	USA	Case-control study (Matched same area)	January 1993 and December 2002	Nonsurgical root canal treated (NSRCT)	196	53.9	-	1-year	✓	✓	✓	✓	-
				Single-tooth implants (STIs)	196	47.5	-	1-year	✓	✓	✓	✓	-
Hannahan, 2008	USA	Cohort study	NR	Root Canal Therapy	143	-	-	1.8 years (1.5 - 4.9)	-	✓	✓	✓	-
				Implant	129	-	-	3 years (1.25–4.75)	-	✓	✓	✓	-
Chatzo-poulos, 2018	USA	Cohort study	2010 to 2016	Root canal treatment (RCT)	8,915	48.89± 17.55	F: 4,552 [51.1] M: 4,363 [48.9]	2.2 ± 1.3 years	✓	-	✓	-	-
								1-year 3-years 5-years	✓	-	-	-	-
				Implant	4,519	60.27± 13.11	F: 2,226 [49.3] M: 2,293 [50.7]	2.8 ± 1.6 years	✓	-	✓	-	-
								1-year 3-years 5-years	✓	-	-	-	-
Vahdati, 2019	USA	Case- control study (Matched same area within same person)	January 1, 2001 to December 31, 2016	NSRCT	170	71.8 [31–97]	F: 85 [50%] M: 85 [50%]	mean: 7.6 years [5–14.2]	✓	✓	✓	✓	-
				STIs	-	-	-	mean: 7.5 years [5–11.7]	✓	✓	✓	✓	-
Hamasha, 2019	Saudi Arabia	Case- control study (Matched by tooth)	NR	RCT-Post-Core	150	41.0	F: 72 [57.1] M: 54 [42.9]	1-year	✓	✓	✓	✓	✓
				Implant	150	44.8	F: 65 [50.4] M: 64 [49.6]	1-year	✓	✓	✓	✓	✓
Esposito, 2020	Italy	Random-ised controlled trial	February 2012 to November 2014	RCT	10	48.6 (39–64)	F: 2 [20.0] M: 8 [80.0]	1-year 3-years 5-years	-	✓	✓	✓	-
				Implant	10	45.2 (34–64)	F: 7 [70.0] M: 3 [30.0]	1-year 3-years 5-years	-	-	✓	✓	-
Lee, 2022	USA	Cohort study	January 1, 1995 to April 30, 2017	STI	321	54.9 ± 14.4 (19, 87)	F: 168 [52.3] M: 153 [47.7]	1-year 3-years 5-years	✓	✓	-	-	-
				Initial non-surgical endodontic treatment (IET)	642	50.3 ± 15.6 (18, 91)	F: 380 [59.2] M: 262 [40.8]	1-year 3-years 5-years	✓	✓	-	-	-
				Non-surgical retreatment (NET)	211	51.3 ± 13.5 (21.83)	F: 155 [73.8] M: 55 [26.2]	1-year 3-years 5-years	✓	✓	-	-	-
				Surgical endodontic retreatment (SET)	79	52.7 ± 13.7 (22.83)	F: 48 [60.8] M: 31 [39.2]	1-year 3-years 5-years	✓	✓	-	-	-
				STI treatments	76	Mean 45.16 ± 9.51	F: 44 [57.9%] M: 32 [42.1%]	5-years	-	✓	-	-	-
Sanz, 2022	Spain	Case-control study (Matched same area within same person)	2017 to 2019	RCT	26	60.65 years (SD 9.93)	F: 16 [61.54%] M: 10 [38.46%]	2-years	-	-	-	-	✓
				Dental implant (DI)	-	-	-	-	-	-	-	-	✓
Gatten, 2011	USA	Cohort study	NR	Single-tooth nonsurgical endodontic therapy	17	-	F: 5 [29.4%] M: 12 [70.6]	1–3.5 years	-	-	-	-	✓
				Single implant-supported prosthesis	20	-	F: 12 [60.0] M: 8 [40.0]	1–6 years	-	-	-	-	✓
Torabinejad, 2014	USA	Cohort study	NR	RCT	24	≥18 years	F: 7 [29.2%] M: 17 [70.8%]	1-year	-	-	-	✓	✓
				Single implant	24	≥18 years	F: 14 [58%] F: 10 [43.0%]	1-year	-	-	-	✓	✓

### Survival, success, and failure rates

Regarding the survival rates, both RCT and DIs showed high survival rates across multiple studies [24, 31, 32, 34] and reported a >95% survival rate for both treatments, having a similar good prognosis. Success rates varied between treatments, with RCT generally showing slightly higher success rates. Two case-control studies [23, 25] reported lower success rates, with 73.5% for DIs and 82.1% for RCT in one study and 75.3% for RCT and 56.7% for DIs in the other. Failure rates ranged from 0.7% to 12% across the studies [23–25, 27, 33, 34], with no significant differences noted, except in a cohort study [34] where implant therapy exhibited significantly lower failure rates than RCT (1.1% vs. 4.1%).

In terms of postoperative complications, implants were associated with more postoperative interventions compared to RCT. One cohort study reported that implants required more postoperative treatments to maintain them despite having similar success rates to RCT [25]. Three case-control studies noted that DI was associated with more postoperative complications than RCT, reported survival with intervention (17.9% vs. 3.6%), reinterventions (12.4% vs. 1.3%), additional treatment (42% vs. 11%), or a number of clinical or technical complications [25, 30]. In the clinical trial [33], three patients in the endodontic group experienced one complication each, compared to a single complication reported in the implant group; this difference was not statistically significant (Table 2).

**Table 2:** Primary outcomes reported in included studies.

Author, year	Treatments	Follow-up time	Clinical/radiological outcomes			
			Survival rate (%)	Succes rate (%)	Failure (%)	Complications
Doyle, 2006	Non-surgical root canal treated (NSRCT)	1-year	8.2	82.1	6.1	Survival with intervention 3.6%
	Single-Tooth Implants (STIs)	1-year	2.6	73.5	6.1	Survival with intervention 17.9%
Hannahan, 2008	Root Canal Therapy	1.8 years (1.5–4.9)	-	99.3	0.7	Reintervention: 1.3%
	Implant	3 years (1.25–4.75)	-	98.4	1.6	Reintervention: 12.4%
Chatzopoulos, 2018	Root canal treatment (RCT)	2.2 ± 1.3 years	95.7	-	4.3	-
		1-year 3-years 5-years	98.3 96.8 95.1			
	Implant	2.8 ± 1.6 years	98.9	-	1.1	-
		1-year 3-years 5-years	99.3 99.1 98.7			
Vahdati, 2019	NSRCT	mean: 7.6 years [5–14.2]	95	94.7	4.7	Additional treatment (%) 11
	STIs	mean: 7.5 years [5–11.7]	95	95.3	5.3	Additional treatment (%) 42
Hamasha, 2019	RCT-Post-Core	1-year	8.7	75.3	12	4.0
	Implant	1-year	24.7	56.7	8.7	10.0
Esposito, 2020	Root canal	1-year 3-years 5-years	-	60.0	10	Fracture (1) Crown de-cementation (1) Moderate pain (1)
	Implant	1-year 3-years 5-years	-	-	0	Abutment screw loosening (1)
Lee, 2022	STI	1-year 3-years 5-years	99.0 99.0 99.0	99.0 98.5 97.2	-	-
	Initial non-surgical endodontic treatment (IET)	1-year 3-years 5-years	96.8 92.1 87.6	96.8 92.1 87.6	-	-
	Non-surgical retreatment (NET)	1-year 3-years 5-years	97.6 90.5 84.4	97.6 90.0 82.1	-	-
	Surgical endodontic retreatment (SET)	1-year 3-years 5-years	92.4 89.5 81.1	92.4 83.4 73.6	-	-
Suganna, 2024	RCT	2-years	93.75	75.57	6.25	-
	Implant	2-years	91.72	80.0	8.27	-
Zang, 2023	NSRCT	5-years	-	88	-	-
	STI treatments	5-years	-	100	-	-
Torabinejad, 2014	RCT	1-year	-	-	-	Number of reported complications (mean [SD]) Day 0: 0.54 [1.587] Day 7: 0.00 [0.000] 3 mo: 0.00 [0.000] 6 mo: 0.00 [0.000] 12 mo: 0.00 [0.000]
	Single Implant	1-year	-	-	-	Number of reported complications (mean [SD]) Day 0: 0.38 [1.135] Day 7: 0.00 [0.000] 3 mo: 0.00 [0.000] 6 mo: 0.00 [0.000] 12 mo: 0.00 [0.000]

### Patient-reported outcomes

Patient-reported outcomes such as pain, satisfaction, and overall QoL were generally similar between the two treatments [24–26, 28]. No significant differences were detected in overall OHIP scores between RCT and DIs, although some dimensions, like physical pain, showed slight differences favouring DI (Table 3).

**Table 3:** Patient-reported outcomes in included studies.

Author, year	Follow-up time	Patient-reported Outcomes		
		Pain	Satisfaction	QoL
Hamasha, 2019	1-year	-	-	OHIP mean improvement = 96.32%
	1-year	-	-	OHIP mean improvement = 89.04%
Sanz, 2022	2-years	Pain during treatment 3.00 (±2.73) Pain after treatment 2.12 (±2.94)	-	OHIP-24 score: 8.82 (15.7)
	-	Pain during treatment 1.93 (±2.33) Pain after treatment 2.00 (±2.51)	-	OHIP-24 score: 7.87 (8.4)
Gatten, 2011	1–3.5 years	-	-	OHIP-14 Mean score: 7.5
	1–6 years	-	-	OHIP-14 Mean score: 3.8
Torabinejad, 2014	1-year	Pain (mean [SD]) VAS Min:0, Max:9 Day 0: 1.67 [3.046] Day 7: 0.17 [0.637] 3 mo: 0.50 [1.615] 6 mo: 0.00 [0.000] 12 mo: 0.00 [0.000]	Satisfaction after treatment (mean [SD]) Questionnaire Min:0, Max:9 Day 7: 8.83 [0.816] 3 mo: 8.63 [1.279] 6 mo: 8.92 [0.408] 12 mo: 8.92 [0.282]	
	1-year	Pain (mean [SD]) VAS Min:0, Max:9 Day 0: 0.38 [0.824] Day 7: 0.50 [1.063] 3 mo: 0.00 [0.000] 6 mo: 0.00 [0.000] 12 mo: 0.00 [0.000]	Satisfaction after treatment (mean [SD]) Questionnaire Min:0, Max:9 Day 7: 8.00 [1.818] 3 mo: 8.46 [0.833] 6 mo: 8.04 [1.601] 12 mo: 8.54 [0.884]	-

## Discussion

This systematic review compared primary and secondary outcomes of RCT and DIs to support evidence-based shared decision-making in dental practice. Overall, no differences were found between the two procedures. Therefore, other factors, such as cost or the impact of edentulism, should guide treatment decisions. Edentulism, particularly in older adults, is associated with adverse outcomes such as depression, malnutrition, and cognitive decline [36–38]. Hakeem et al. found that each missing tooth increases frailty risk in individuals aged 60 years and older. These considerations are important when deciding whether to extract a natural tooth [39].

Dentists should provide patients with detailed and objective information on each treatment option – including costs, success and failure rates, complications, and patient-reported outcomes like pain and QoL – to enable shared decision-making. Despite the relevance of these issues, few studies have directly compared outcomes between RCT and DIs.

Both treatments demonstrated high survival rates. One study reported a 95% survival rate at 5 years, while another found slightly higher DI survival at 3 years (98.7% vs. 95.1%) [24]. Another study reported 99% DI survival and 87.6% RCT survival at 5 years [32]. However, inconsistencies in survival definitions – such as including teeth with unhealed or uncertain status [23], or using different criteria like absence of mobility or radiolucency [31] – make comparisons challenging. These variations notwithstanding, both treatments generally showed strong survival rates.

Success rates also varied across studies because of differing definitions. Most defined success as the absence of reintervention, often supplemented by radiographic criteria such as the absence of radiolucency (RCT) or marginal bone loss ≤4 mm (DIs) [35]. Lee et al. found DIs had higher clinical (97.2% vs. 83.7%) and radiological success (96.4% vs. 81.2%) at 5 years [32]. Zang et al. also favoured DIs (100% vs. 88%) [35], whereas Doyle and Hamasha found better outcomes for RCT (75.3%–82.1% vs. 56.7%–73.5%) [23, 25]. Some studies found similar success rates for both [24, 27], though factors like periodontal disease and caries influenced DI outcomes. Suganna et al. reported no significant difference, noting that overlapping definitions of success and survival complicate comparisons [31]. Thus, success rates depend largely on study definitions and clinician expertise.

Failure rates were low and comparable across treatments. Failure was generally defined as the removal or planned removal of the treated tooth or implant. Causes of failure included prosthetic, periodontal and endodontic issues [23], infections, foreign body reactions [27], loss of osseointegration, and fractures [34]. Risk factors such as age, anxiety [34], systemic disease, smoking, and bone density influenced outcomes in both procedures. Clinician skills remain critical to minimising failure risk.

Complications varied in type and frequency. DI was associated with more reinterventions. Hannahan et al. reported 12.4% for DIs versus 1.3% for RCT [27], and Vahdati et al. reported 42.0% for DIs versus 11.0% for RCT [24]. RCT complications included caries, procedural mishaps, flare-ups, and fractures [24, 30, 33]. DI complications involved more frequent medication use (100% vs. 2%) [24], abutment loosening [33], sensory disturbances, bone loss, and esthetic or mechanical issues [24]. Despite these, patient-reported satisfaction and perceived complication rates remained high for both treatments [24]. Although direct comparison is difficult due to heterogeneous reporting, complication rates were low and should be discussed with patients during treatment planning.

Patient-related outcomes – including pain, satisfaction, and QoL – were favourable for both treatments. QoL was assessed using the OHIP, particularly the OHIP-14 scale [9], which showed notable improvements after both procedures [24, 25, 28]. Hamasha et al. found that gender influenced perceived improvement, with women reporting more significant gains [25]. Pain levels were minimal across both treatments in the short and long term [26, 30], and no significant differences in satisfaction were found [30]. While RCT may cause more procedural discomfort, overall patient experiences were positive and comparable.

This review should be interpreted with caution because of certain limitations. Inconsistencies in outcome definitions and follow-up durations limited direct comparisons. Study quality varied, with issues such as incomplete confounding control, loss to follow-up, and insufficient baseline data. While procedures were conducted by trained clinicians, many studies lacked details on prognostic factors like systemic health, radiographic findings, and patient preferences. Some included studies were older and may not reflect current clinical practices. In addition, most studies were from the USA [23, 24, 27, 28, 30, 32, 34], China [35], Spain [26], India [31], Italy [33], and Saudi Arabia [25]. The lack of data from Latin America, Africa, or Oceania may limit generalisability due to cultural, socioeconomic, and healthcare disparities.

A key limitation of this review is the reliance on observational studies, which are prone to bias because of lack of randomisation. For instance, patients with less severe cases may have been more likely to receive RCT, while more complex cases were directed to implant treatment, introducing selection bias. In addition, uncontrolled confounders such as patient health status or clinician experience may affect outcomes and limit the strength of causal conclusions.

Despite these limitations, this review contributes meaningfully to the existing literature. It builds upon earlier systematic reviews [40–42], including Torabinejad et al., which compared outcomes of RCT, DIs, fixed prostheses, and extraction without replacement [43]. A more recent review done by Sinsareekul et al. included eight observational studies and found mixed results: some reported no difference in short-term survival, while others found higher DI survival or fewer complications [40]. Patient satisfaction remained high across treatments.

Future research should standardise definitions of success and failure, and adopt prospective designs with clear follow-up criteria [44]. Studies should also assess periodontal and peri-implant health, as well as patient-level risk factors such as age, systemic disease, and lifestyle. Investigating long-term effects in diverse populations will enhance the generalisability of findings [45]. Personalising treatment by considering individual health status, gender, and preferences can further improve outcomes [38, 44, 46]. Lastly, incorporating cost-effectiveness analyses would aid in balancing clinical benefits with patient and systemic financial constraints [18, 35, 47, 48].

## Conclusions

This systematic review found that both RCT and DIs are effective treatment options for managing severely compromised teeth, demonstrating high survival and success rates, alongside low complication and failure rates. Importantly, both treatments were also associated with favourable patient-reported outcomes, including low levels of pain, high satisfaction, and comparable improvements in QoL. These findings support the importance of incorporating patient-centred outcomes into treatment planning. The choice between treatments should be guided by a thorough assessment of individual clinical factors, patient preferences, anticipated long-term outcomes, and economic considerations. Future research should incorporate more diverse populations and emphasise standardised definitions, patient-centred outcomes, and cost-effectiveness analyses to enhance evidence-based clinical decision-making.

## Supplementary Material



## Review registration

The protocol was registered on the International Prospective Register of Systematic Reviews (PROSPERO) No. CRD42024584113. As part of the review process, the original search strategy defined in the PROSPERO-registered protocol was updated in February 2025. In addition to the databases originally specified (MEDLINE, EMBASE, LILACS, and The Cochrane Library), the search was expanded to include Scopus, Web of Science, BASE (Bielefeld Academic Search Engine), and Google Scholar. This update aimed to ensure a more comprehensive identification of relevant studies and minimise publication bias.
